# Characterization of a recently synthesized microtubule-targeting compound that disrupts mitotic spindle poles in human cells

**DOI:** 10.1038/s41598-021-03076-3

**Published:** 2021-12-08

**Authors:** Dilan Boodhai Jaunky, Kevin Larocque, Mathieu C. Husser, Jiang Tian Liu, Pat Forgione, Alisa Piekny

**Affiliations:** 1 Department of Biology, Concordia University, Montreal, QC Canada; 2 Department of Chemistry and Biochemistry, Concordia University, Montreal, QC Canada

**Keywords:** Centrosome, Microtubules, Cytoskeleton, Cell division, Mitosis, Mitotic spindle

## Abstract

We reveal the effects of a new microtubule-destabilizing compound in human cells. C75 has a core thienoisoquinoline scaffold with several functional groups amenable to modification. Previously we found that sub micromolar concentrations of C75 caused cytotoxicity. We also found that C75 inhibited microtubule polymerization and competed with colchicine for tubulin-binding in vitro. However, here we found that the two compounds synergized suggesting differences in their mechanism of action. Indeed, live imaging revealed that C75 causes different spindle phenotypes compared to colchicine. Spindles remained bipolar and collapsed after colchicine treatment, while C75 caused bipolar spindles to become multipolar. Importantly, microtubules rapidly disappeared after C75-treatment, but then grew back unevenly and from multiple poles. The C75 spindle phenotype is reminiscent of phenotypes caused by depletion of ch-TOG, a microtubule polymerase, suggesting that C75 blocks microtubule polymerization in metaphase cells. C75 also caused an increase in the number of spindle poles in paclitaxel-treated cells, and combining low amounts of C75 and paclitaxel caused greater regression of multicellular tumour spheroids compared to each compound on their own. These findings warrant further exploration of C75’s anti-cancer potential.

## Introduction

Dynamic microtubules are required to form mitotic spindles, and drugs that suppress these dynamics are used to treat cancer^1,2^. At the G2/M transition, there is an increase in microtubule growth from the maturing centrosomes. Dimers of α-tubulin and β-tubulin form 13 protofilaments that roll into a tubule, which are templated by the γ-tubulin ring complex^3,4^. GTP-bound dimers at the plus end promote microtubule growth, while the loss of this cap favors catastrophe^4–6^. During catastrophe the protofilaments adopt a curved state and bend away from the lumen^7,8^. Microtubule dynamics are required to form stable kinetochore attachments, and many microtubule-associated proteins (MAPs) influence these dynamics by stabilizing minus or plus ends^9^. MAPs include enzymes such as CKAP5/ch-TOG and MCAK that control polymerization and depolymerization, respectively, for bipolar spindle assembly^5,10–16^. Microtubule-targeting drugs that suppress microtubule dynamics and prevent the formation of stable kinetochore attachments can lead to cell cycle arrest or catastrophe, and cell death.

Several microtubule-targeting drugs are currently being used to treat cancers. Uncontrolled cell proliferation is a hallmark of cancer, which makes cancer cells more sensitive to drugs that disrupt the cell cycle compared to healthy cells^17,18^. Taxol™ is a microtubule-targeting compound that is used to treat several cancers^19^. Taxol binds to microtubules and stabilizes the lattice to prevent depolymerization^20,21^. These reduced dynamics disrupt the formation of stable kinetochore attachments triggering the spindle assembly checkpoint to cause cell cycle arrest or mitotic catastrophe^22^. Although Taxol has been successful in the clinic, it causes side-effects and some patients develop resistance^23^. Thus, there is a need to find alternative drugs that can reduce the amount of Taxol needed for treatment, reducing side-effects and resistance.

Other microtubule-targeting compounds destabilize microtubules. These altered dynamics also cause mitotic arrest or catastrophe and have been used or considered for use as anti-cancer therapies. One of these compounds is colchicine, which binds to a deep pocket on β-tubulin and prevents growth or causes catastrophe at the plus-end and has a low off-rate^24–27^. Colchicine is used to treat gout, but failed as an anti-cancer therapy^28,29^. Another compound is vinblastine, which is used as an anti-cancer drug^8,30^. It binds to the interface of the heterodimer and introduces a molecular wedge causing the protofilaments to adopt a curled conformation that destabilizes the microtubule^8,29^. Vinblastine has fewer side-effects compared to colchicine. In addition to having distinct binding sites, they have different properties in vivo, including accessibility to the minus vs. plus ends and how they affect microtubule polymers^8^. In vitro, vinblastine favours depolymerization at the minus ends and suppresses dynamics at the plus ends^31^. In HeLa cells, low concentrations (e.g. 2 nM) do not cause changes in the microtubule polymer mass, but block mitosis by decreasing kinetochore attachments, and causing the dissociation of mother and daughter centrioles^32,33^.

Cancer cells often accumulate mutations that alter the expression or function of MAPs, which can increase their sensitivity to microtubule-targeting drugs^1,34^. Coupling MCAK depletion with low concentrations of Taxol or vinblastine in cancer cells increases mitotic spindle phenotypes and causes apoptosis^35^. Aurora A kinase, which regulates centrosome maturation and plus-ends for kinetochore attachments, and ch-TOG (CKAP5), a microtubule polymerase, are over-expressed in colorectal cancer cells^14,36–42^. Their overexpression correlates with increased rates of microtubule assembly and chromosomal instability (CIN) due to an excess of lagging chromosomes^37,42^. These properties could make mitotic spindles sensitive to further disruption by microtubule-targeting drugs compared to healthy cells.

We recently synthesized a novel family of theionoisoquinoline compounds that target microtubules. We strategically designed the compounds to have properties ideal for in vivo use and screened derivatives for their ability to block cancer cell proliferation. The compounds share a core scaffold amenable to modifications^43^. We identified several derivatives that caused toxicity and mitotic arrest with IC_50_ values in the sub micromolar range^44^. Some derivatives had no or little effect on cells (e.g. C87, IC_50_ > 10 uM) while others, such as C75, had high efficacy (e.g. IC_50_ between 0.1 and 0.4 uM) depending on the cell type^44^. We also found that C75 prevented microtubule polymerization and competed with colchicine for tubulin-binding in vitro^44^.

Here, we characterized the phenotypes caused by C75 to elucidate its mechanism of action in cells. While longer treatments of C75 or colchicine caused similar mitotic spindle phenotypes, the two compounds synergistically increased cytotoxicity, supporting differences in how they affect microtubules in cells. Indeed, colchicine and C75 caused different spindle phenotypes. When added in metaphase, spindles remained bipolar and eventually collapsed after colchicine treatment, while C75 caused bipolar spindles to become disorganized and/or multipolar. C75 phenocopies ch-TOG depletion suggesting that it blocks microtubule polymerization in cells. In addition, C75 caused an increase in the number of spindle poles in cells where microtubules were stabilized by paclitaxel. Excitingly, combining low doses of C75 and paclitaxel caused greater regression of multicellular tumour spheroids compared to each compound on their own, warranting further exploration of C75 as an anti-cancer drug.

## Results

### Compound 75 causes cells to arrest in G2/M phase

**Figure 1:**
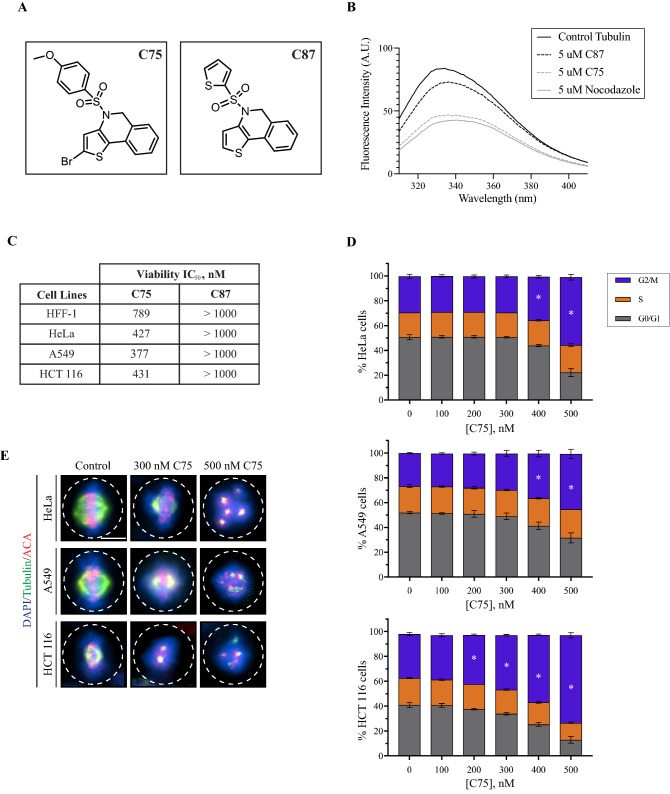
C75 is a thienoisoquinoline compound that causes G2/M arrest in cultured human cells. (**A**) The structures of C75 and C87, a derivative with minimal activity, are shown. (**B)** A graph shows the absorbance of tubulin (control, grey line) measured by a change in fluorescence (y-axis), compared to when 5 uM of C87, C75 or nocodazole was added to tubulin for the wavelengths as shown on the x-axis. (**C)** A table shows the IC_50_ for the viability of HFF-1, HeLa, A549 and HCT 116 over three population doubling times after treatment with C75 or C87 (N = 3). (**D)** Bar graphs show the distribution of cells in G0/G1, S and G2/M phases of the cell cycle measured by flow cytometry, for HeLa, A549 and HCT116 cells treated with increasing concentrations of C75 for 8 h (n = 20,000 cells per treatment; N = 3 experimental replicates). Asterisks indicate statistical significance using two-way ANOVA test and post-hoc Tukey’s multiple comparison test with a 95% CI, multiplicity adjusted *p* =  < 0.0004. (**E)** Images show fixed HeLa, A549 and HCT116 cells immunostained for DNA (DAPI; blue), tubulin (green), and centromeres (ACA; red) after treatment with 300 or 500 nM of C75 for 8 h. Dotted lines outline the cells. The scale bar is 10 µm.

C75 is a new microtubule-targeting compound, and we wanted to characterize its mechanism of action in cultured human cells. The structure of C75 is shown in Fig. 1A along with C87, an inactive derivative. We previously found that C75 competes for colchicine binding to tubulin in vitro^44^. Since this competition could be indirect, we determined if C75 directly binds to tubulin. By monitoring changes in the emission spectra of tubulin, we found that C75 or nocodazole, another microtubule-depolymerizing compound, caused a similar decrease in emission compared to DMSO (control) or C87 (Fig. 1B). C75 was identified by structure–activity-relationship studies assaying for cytotoxic effects on A549 cells^44^. We found that C75 also caused toxicity in other cell types. The IC_50_ in HFF-1 (male foreskin fibroblast), HeLa (female cervical adenocarcinoma), A549 (male lung carcinoma), and HCT 116 (male colorectal carcinoma) cells was 789, 427, 377 and 431 nM, respectively, while there was little/no toxicity for C87 (Fig. 1C). This data shows that C75 has ~ twofold higher sensitivity for some cell types compared to others. To determine if the reduced cell viability is due to cell cycle defects, we performed flow cytometry on HeLa, A549 and HCT 116 cells after treatment with increasing concentrations of C75 for 8 h. There was a significant increase in the proportion of cells in G2/M with 400 or 500 nM C75 in all three cell lines with a corresponding decrease in G1 (Fig. 1D, S1A). We also observed significant changes in the proportion of cells in G2/M and G1 in HCT 116 cells after treatment with 200 and 300 nM C75 (Fig. 1D, S1A). In contrast, the proportion of HeLa and A549 cells in S phase remained the same and changed only in HCT 116 cells after treatment with 500 nM C75 (Fig. 1D, S1A).

**Figure 2:**
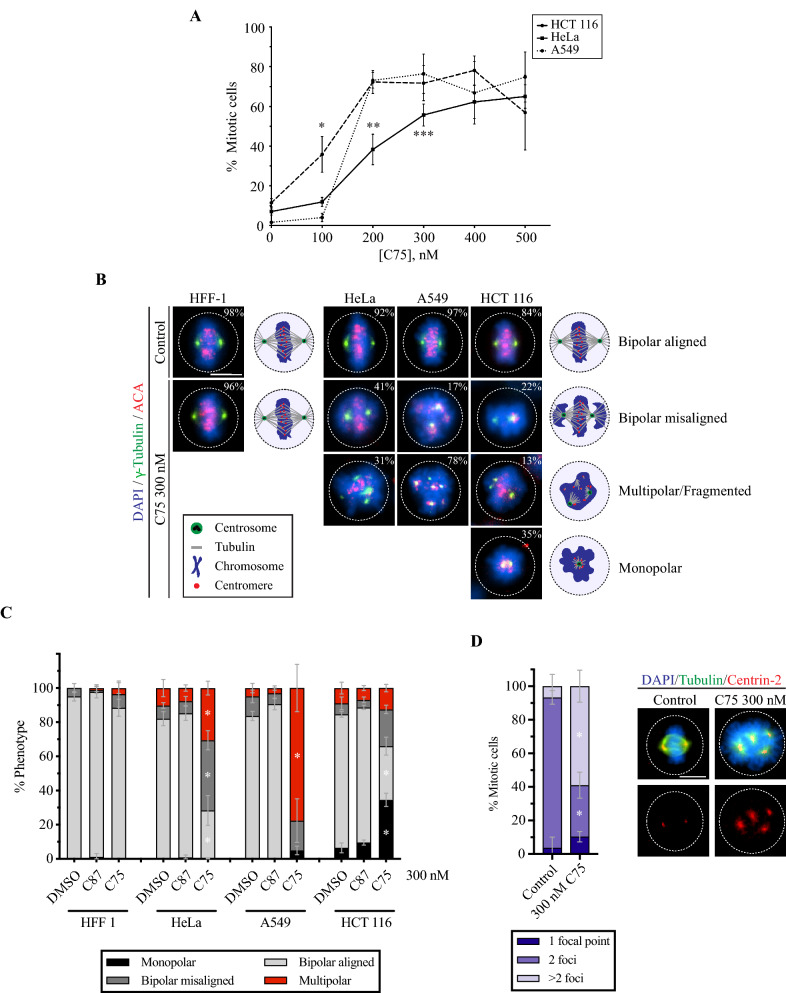
C75 causes the formation of multipolar spindles and mitotic arrest. (**A**) A line graph shows changes in the proportion of mitotic HeLa, A549 and HCT 116 cells after treatment with increasing concentrations of C75 for 24 h. Asterisks indicate statistical significance using a two-way ANOVA test and post-hoc Tukey’s multiple comparison test with a 99% CI; a single asterisk means that HCT 116 is significantly different than HeLa and A549 (multiplicity adjusted *p* = 0.0007 and *p* < 0.0001, respectively), two indicates that HeLa is significantly different than A549 and HCT 116 (multiplicity adjusted *p* < 0.0001 for both), and three indicates that HeLa is significantly different than A549 (multiplicity adjusted *p* = 0.0035). (**B**) Images show fixed HFF 1, HeLa, A549 and HCT116 cells stained for DNA (DAPI; blue), g-tubulin (green), and centromeres (ACA; red) after treatment with 300 nM of C75 for 4 h. Cartoon schematics (green circles, centrosomes; blue, chromatin; grey lines, microtubules; red, centromeres) show the different phenotypes observed, including bipolar spindles with aligned chromosomes (top), bipolar spindles with misaligned chromosomes (second from top), multipolar spindles (third from top), or monopolar spindles (bottom). The proportion of cells with each phenotype is shown in the top right corner of each image. The scale bar is 10 µm. (**C**) A bar graph shows the proportion of each spindle phenotype (bipolar aligned in light grey; bipolar misaligned in dark grey; multipolar in red; monopolar in black) for HFF 1, HeLa, A549 and HCT 116 cells treated as in **B**). Bars show standard deviation and asterisks indicate two-way ANOVA test and a post-hoc Tukey’s multiple comparison test with a 99% CI, and multiplicity adjusted *p* =  < 0.001 for each phenotype vs. DMSO and C87. (**D**) A bar graph shows the proportion of HeLa cells with 1 (dark purple), 2 (purple) or more (light purple) centrin-2 foci after treatment with DMSO or 300 nM C75. Cells were fixed and stained for DNA (DAPI; blue), tubulin (green), and centrin-2 (red). Asterisks indicate statistical significance of C75 with 2 foci and > 2 foci compared to DMSO using multiple t test with a 95% CI with a multiplicity adjusted *p* = 0.0009 and *p* = 0.0031, respectively. The scale bar is 10 µm.

The increase in G2/M cells caused by C75 implies mitotic delays or arrest. To assess mitotic phenotypes, cells were fixed and stained for DNA, microtubules and centromeres after treatment with 300 or 500 nM C75. While control HeLa, A549 and HCT 116 metaphase cells had bipolar spindles and aligned chromosomes, treated cells had spindles with reduced microtubule intensity, misaligned chromosomes and multiple spindle poles (Fig. 1E). We also measured the proportion of rounded, mitotic HeLa, A549 and HCT 116 cells 24 h after treatment with varying concentrations of C75 (Fig. 2A). We saw a dramatic increase in the proportion of mitotic cells at 300 nM (55.7% HeLa, 76.4% A549 and 71.7% HCT 116, respectively). Therefore, increasing concentrations of C75 appear to cause mitotic arrest in different cell types. To determine the fate of C75-treated HeLa cells, we followed individual cells for ~ 17 h by live imaging. While control cells underwent mitosis as expected, C75-treated cells failed to exit mitosis and underwent apoptosis (Figure S1B).

### C75 causes spindle phenotypes

We propose that C75 arrests cells in mitosis by causing spindle defects. Since our previous experiments were done over long periods of time (e.g. 8–24 h), we quantified spindle phenotypes after treatment for less time to capture a broader range of phenotypes. HFF-1, HeLa, A549 and HCT 116 cells were treated with 300 nM C75 for 4 h and stained for DNA, γ-tubulin and centromeres (Fig. 2B). While spindles in C75-treated HFF-1 cells were similar to control, HeLa, A549 and HCT 116 cells had monopolar (centrosomes failed to separate), bipolar misaligned (misaligned sister chromatids) and multipolar spindles (Fig. 2B,C). HeLa cells had a significant increase in bipolar misaligned (41.1 vs. 7.1% control) and multipolar spindles (30.6 vs. 7.6% control), while A549 cells had an increase in monopolar (5 vs. 0% control) and multipolar spindles (77.6 vs. 3% control) and HCT 116 cells had monopolar (34.5 vs. 9.5% control), bipolar misaligned (21.5 vs. 4.4% control) and multipolar spindles (12.5 vs. 6.9% control; Fig. 2C). The different proportions of spindle phenotypes caused by C75 in each cell line likely reflects differences in their genetic backgrounds.

To determine if other centrosome components localize to the poles of multipolar spindles, we repeated this experiment using HeLa cells and stained for DNA, tubulin and centrin-2, which marks centrioles (Fig. 2D). Since individual centrioles were difficult to resolve by light microscopy, we quantified the number of foci and assumed that each contains a minimum of two centriole pairs. Indeed, a significantly greater proportion of C75-treated cells had more than 1 or 2 centrin-2 foci compared to control cells (59 vs. 10%). Therefore, both γ-tubulin and centrin-2 are localized to multipolar spindles in C75-treated cells.

### Combining C75 and colchicine increases spindle phenotypes

**Figure 3:**
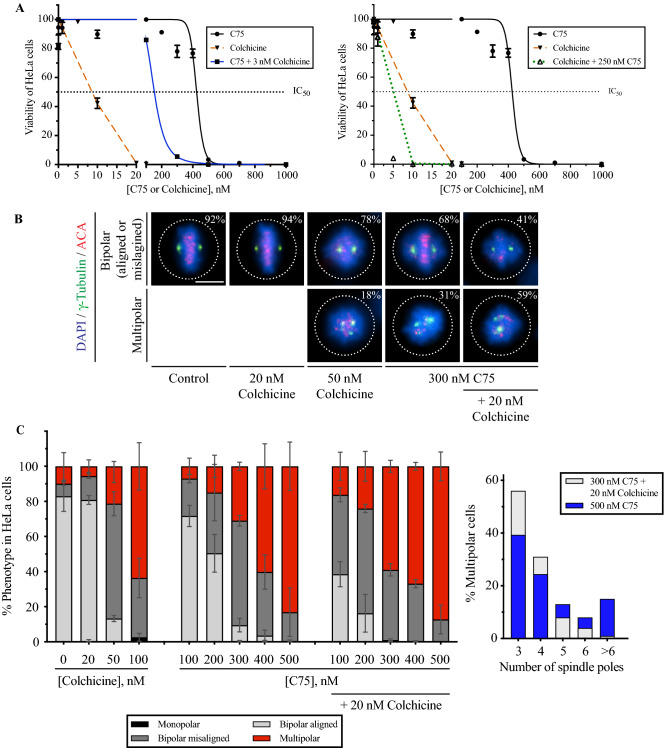
Combining C75 with colchicine enhances lethality and increases spindle phenotypes. (**A**) Line graphs show the IC_50_ for the viability (dotted black lines) of HeLa cells treated with varying concentrations of C75, colchicine, or both after three population doubling times as indicated. The graph on the left shows changes in viability (Y-axis) with increasing concentrations of C75 (X-axis; black line), colchicine (dotted orange line), and C75 with 3 nM colchicine (blue line). The graph on the right shows the changes in viability with colchicine and 250 nM C75 (green dotted line). The bars indicate SEM for N = 3 experimental replicates. (**B)** Images show fixed HeLa cells stained for DNA (DAPI; blue), g-tubulin (green) and ACA (centromeres; red) after treatment for 5 h with colchicine, C75 or both. The proportion of cells with bipolar spindles (aligned or misaligned chromosomes) and multipolar spindles are indicated on the images. The scale bar is 10 µm. (**C)** Bar graphs show the proportion of HeLa cells from B) with bipolar aligned (light grey), bipolar misaligned (dark grey), multipolar (red) or monopolar (black) spindles. Bars show standard deviation. Statistical analysis was done using two-way ANOVA test and a post-hoc Tukey’s multiple comparison test with a 90% CI, and multiplicity adjusted *p* =  < 0.0831; a single asterisk indicates significance to the control (DMSO) and two indicates significance to C75. (**D)** A bar graph shows the percentage of multipolar HeLa cells from (**C**) with different numbers of spindle poles after treatment with 300 nM C75 plus 20 nM colchicine (light grey) or 500 nM C75 (blue).

Mitotic spindle phenotypes have been previously reported for colchicine^24^. Although C75 competed with colchicine for tubulin-binding in vitro, they could bind to different sites or have different accessibility to microtubules in cells. In this case, we expect to see additive or synergistic interactions, which we tested using toxicity assays and comparing the IC_50_ for each compound alone vs. adding them together. The IC_50_ for C75 and colchicine in HeLa cells was 425 and 9.5 nM, respectively (Fig. 3A). Adding 3 nM colchicine, a sub-optimal concentration that had little effect on viability, lowered the IC_50_ of C75 to 153 nM (Fig. 3A). Similarly, adding 250 nM of C75 to colchicine lowered its IC_50_ to 1.9 nM (Fig. 3A). To ascertain whether the combinatorial treatments were synergistic, additive or antagonistic, we analyzed the data with CompuSyn (https://www.combosyn.com/index.html)^45^. Using the non-constant ratio method, we determined the Combination Index (CI) for each drug combination and found synergies with 3 nM of colchicine and 300 nM of C75 (CI = 0.20), and when 250 nM C75 was combined with 5 nM colchicine (CI = 0.25; Fig. S2A).

Next, we measured changes in the spindle phenotypes caused by combining the two compounds. HeLa cells were treated for 4 h with 20 or 50 nM colchicine, and 100, 200 or 300 nM of C75 with or without 20 nM colchicine. Cells were fixed and stained for DNA, γ-tubulin and centromeres, and the proportion of cells with bipolar, bipolar misaligned or multipolar spindles was counted for each treatment (Fig. 3B,C). While cells treated with DMSO (control), 20 nM colchicine, 100 or 200 nM C75 had similar proportions of bipolar misaligned or multipolar spindles, higher concentrations of colchicine (50 nM) or C75 (300 nM) caused an increase in spindle phenotypes (Fig. 3B,C). Adding 20 nM of colchicine to 300 nM C75 caused a significant increase in the proportion of cells with multipolar spindles beyond the sum of each compound on their own at those concentrations (Fig. 3B,C). Therefore, C75 and colchicine enhance each other at sub-lethal concentrations. We also determined if combining C75 and colchicine causes more severe multipolar spindle phenotypes. While the number of spindle poles in multipolar cells was similar for cells treated with 300 nM C75, 50 nM colchicine or 300 nM C75 with 20 nM colchicine, cells treated with 500 nM of C75 had a greater proportion of cells with six or more poles (Fig. 3D, also Fig. S2B). This data suggests that colchicine and C75 have different effects on spindles.

### C75 and colchicine have different effects on mitotic spindles in cells

**Figure 4:**
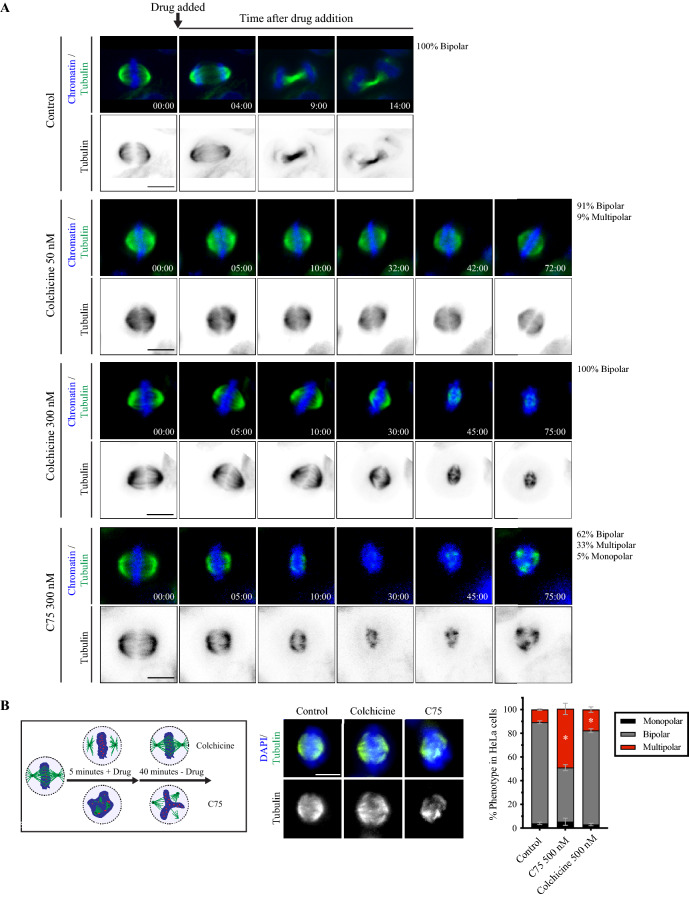
C75 and colchicine cause different spindle phenotypes. (**A**) Timelapse images show live HeLa cells co-stained for DNA (Hoechst 33,342; blue) and microtubules (SiR-tubulin; green). Times are indicated in minutes. An arrow points to the time of addition of DMSO (control; n = 15), 50 nM colchicine (n = 33), 300 nM colchicine (n = 20) or 300 nM of C75 (n = 21) to cells. The proportion of cells with bipolar, multipolar or monopolar spindles are indicated. (**B)** A cartoon schematic shows how HeLa cells were treated with drug (DMSO, 500 nM C75 or colchicine) for 5 min, then fixed 40 min after the drugs were washed out. Underneath, images show fixed HeLa cells co-stained for DNA (DAPI; blue) and tubulin (green or white) after treatment with C75 or colchicine. To the right, a bar graph shows the proportion of cells with monopolar (black), bipolar (dark grey) or multipolar (red) spindles. Bars show standard deviation and asterisks indicate *p* < 0.05 by multiple t tests vs. control. The scale bar for all cells is 10 µm.

Next, we compared the spindle phenotypes caused by C75 and colchicine. We performed live imaging to determine the effects of C75 and colchicine on mitotic spindles. HeLa cells were pre-treated with Hoechst 33,342 and SiR-tubulin to visualize chromatin and microtubules, respectively. Cells with bipolar spindles and aligned chromosomes were treated with 50 nM or 300 nM colchicine, or 300 nM C75 and imaged for changes in spindle morphology. The majority of control cells (DMSO) had bipolar spindles that matured to segregate sister chromatids and form a midbody (14/15), while one cell had a tripolar spindle (Fig. 4A). The majority of cells treated with 50 nM colchicine had bipolar spindles that arrested (30/33), although a small proportion had multipolar spindles (3/33; Fig. 4A). Cells treated with 300 nM colchicine also had bipolar spindles that arrested (20/20), and 5/20 had extensive microtubule loss and collapsed spindles (Fig. 4A). Cells treated with 300 nM C75 showed a mix of phenotypes including arrested bipolar spindles with reduced microtubules (12/21), multipolar spindles (7/21) and a monopolar spindle (1/21; Fig. 4A). We were surprised to see that microtubules regrew in C75-treated cells, which was not observed in any of the colchicine-treated cells (Fig. 4A). Thus, C75 and colchicine cause different spindle phenotypes, with the caveat that few concentrations were tested.

Next, we compared how mitotic spindles recover after release from short-term treatments. HeLa cells were treated with 500 nM of C75 or colchicine for 5 min, then the drugs were washed out, and cells were left to recover for 40 min prior to fixing and staining them for DNA and tubulin (Fig. 4B). With this time frame, cells could have been in late G2 or early mitosis upon treatment. A larger proportion of C75-treated cells had multipolar spindles (49.7 vs. 45.7% bipolar) compared to those treated with colchicine (17.7 vs. 79.2% bipolar; Fig. 4B). The small, but significant increase in multipolar spindles after colchicine treatment suggests that multipolar spindles arise from effects caused prior to metaphase.

Next, we determined if C75 and colchicine cause spindle phenotypes when added prior to mitosis. HeLa cells stably expressing GFP- or mCherry-tagged tubulin were imaged entering mitosis after treatment with 50 nM colchicine or 300 nM C75 in late S or G2 phase (Fig. S3A,B). Control cells entered mitosis with bipolar spindles that progressed to telophase (12/12, Fig. S3A; 22/22, Fig. S3B). We observed multiple centrosomes clustering to form bipolar spindles in many control cells (e.g. Fig. S3B). Colchicine caused multipolar spindles to form without transitioning through a bipolar state in 24% of cells (18/74), while only 9% of C75-treated cells (4/44) had multipolar spindles (Fig. S3A,B). This data shows that the multipolar spindle phenotypes observed after longer colchicine treatments arise prior to mitosis, while the multipolar spindles caused by C75 occur in metaphase, after bipolar spindles have already formed.

### Spindle microtubules regrow in the presence of C75

**Figure 5:**
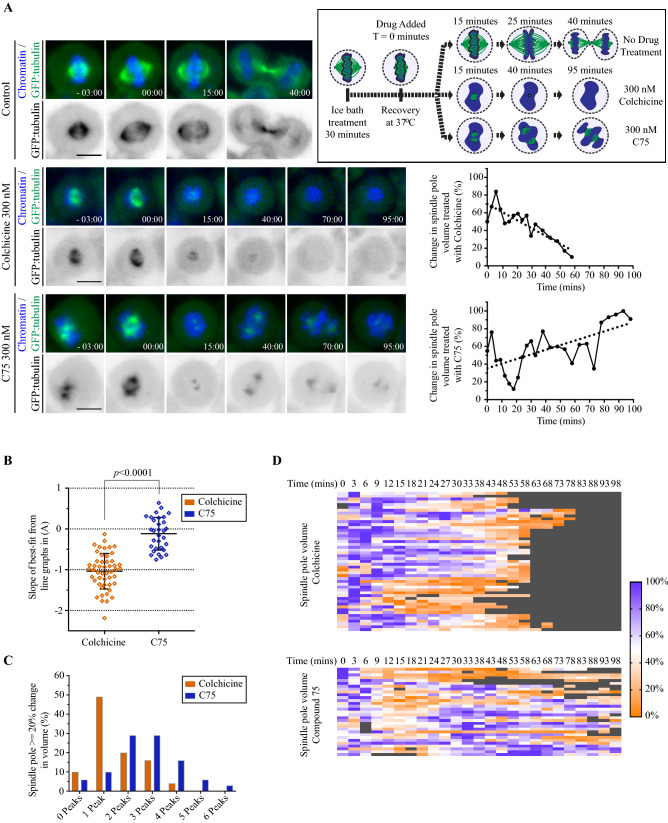
Microtubules recover in the presence of C75. (**A**) A cartoon schematic (box at upper right) shows the experimental design. HeLa cells were cold-treated for 30 min in an ice bath to collapse microtubules, then upshifted to 37 °C and imaged for microtubule regrowth in the presence or absence of 300 nM colchicine or C75. Timelapse images show live HeLa cells stably expressing GFP:tubulin (green) and co-stained for DNA by Hoechst (blue), with the times indicated in minutes. DMSO (control, n = 6), 300 nM colchicine (n = 24) or 300 nM C75 (n = 15) was added at 0 min. To the right, line graphs show changes in spindle pole volume (%; Y-axis) over time (X-axis) after addition of colchicine or C75 (dotted line indicates best-fit). (**B)** A scatter plot shows the distribution of slope values obtained from best-fit line graphs (Y-axis) for the changes in each spindle pole volume over time after treatment with C75 or colchicine as in A). Significance was determined using the two-tailed Welch’s t test, *p < *0.0001. (**C)** A bar graph shows the number of peaks (X-axis) with an amplitude equal to or greater than 20% of the maximum volume of the spindle pole after treatment with C75 or colchicine (Y-axis). (**D)** Heat maps show the change in volume (%) of individual spindle poles over time (minutes, at the top) in cells treated with colchicine or C75 as in A). Purple indicates larger volumes while orange indicates smaller volumes. Each line represents a different spindle pole.

To further compare the effect of C75 and colchicine on microtubules in cells, we monitored the re-growth of microtubules in the presence of the two compounds. HeLa cells stably expressing GFP-tagged tubulin were cold-treated for 30 min to depolymerize microtubules, then 300 nM C75 or colchicine was added upon upshift to 37 °C, noting that the temperature upshift took a few minutes. Cells with bipolar spindles were imaged during recovery as shown in Fig. 5A. All of the control cells formed functional spindles and were in telophase by ~ 40 min. In colchicine-treated cells, microtubules decreased with no detectable GFP signal by ~ 50 min, as expected given its low off-rate (24/24; Fig. 5A). In C75-treated cells, microtubules also decreased, but GFP regained intensity as microtubules regrew to some extent in all cells (15/15; Fig. 5A). To further characterize these phenotypes, we quantified changes in spindle pole volume and maximum intensity over time (Figs. 5A–D, S4A,B). The spindle poles were identified as spherical objects in 3D-reconstructed cells in Imaris v.9.7.2 (Bitplane), which were tracked and quantified over time for changes in volume and intensity. Colchicine-treatment caused a decrease in the volume and maximum intensity of spindle poles over time (Figs. 5A–D, S4A,B). While some fluctuation was observed, there was a net linear decrease in volume and intensity over time as indicated by the best-line fits with negative slopes (Figs. 5A,B, S4A,B). Heatmaps of the proportional changes in spindle volume or intensity show how each pole decreased over time, albeit some sooner than others (purple to orange; Figs. 5D, S5B). However, C75-treatment caused an initial decrease in spindle volume or intensity followed by recovery in multiple cells (Figs. 5A,B, S4A,B). This was evident in the heatmaps where spindle poles continued to recover and fluctuate over time (alternating orange and purple; Figs. 5D, S4B). To better understand this oscillatory pattern, we quantified the number of peaks where there was a > 20% change in pole volume (Fig. 5C). More poles in C75-treated cells had > 2 peaks (54 vs. 46% with 1 or 2) compared to colchicine-treated cells (31 vs. 69% with 1 or 2; Fig. 5C). Thus, microtubules can polymerize in the presence of C75, but this growth is not evenly distributed between the poles. This data suggests that C75 could have a high off-rate and/or a limited window of accessibility to microtubules.

### The C75 phenotype is reminiscent of a disruption in microtubule polymerization

**Figure 6:**
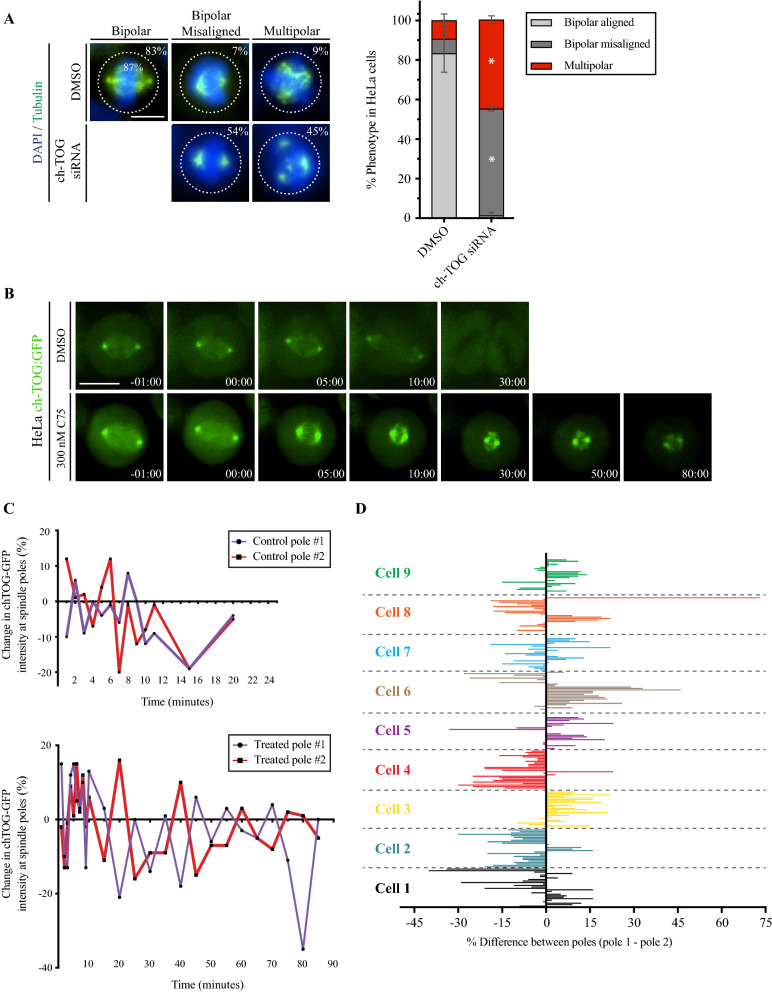
C75 causes a change in ch-TOG localization. (**A**) Images show fixed HeLa cells stained for DNA (DAPI, blue) and microtubules (tubulin, green) after treatment with ch-TOG siRNAs. The proportion of cells with bipolar spindles and aligned chromosomes, bipolar spindles with misaligned chromosomes, or multipolar spindles is indicated on the images. The scale bar is 10 µm. A bar graph shows the proportion of cells (%) with bipolar spindles (light grey), bipolar spindles with misaligned chromosomes (dark grey) or multipolar spindles (red). Bars show standard deviation. Asterisks show *p* < 0.0001 with respect to control (DMSO) using two-way ANOVA with a 99% confidence interval. (**B)** Timelapse images show live HeLa cells expressing ch-TOG:GFP (green) upon addition of DMSO (control, n = 13) or 300 nM C75 (n = 10). Times are indicated in minutes. The scale bar for all cells is 10 µm. (**C)** Line graphs show the percent change in ch-TOG:GFP signal intensity (Y-axis) over time (minutes, X-axis) for each pole in an individual control or C75-treated cell. (**D)** A bar graph shows the relative change in proportion of ch-TOG from pole 1 to pole 2 (arbitrarily chosen) over time (each bar is 1 min for the first 10 min, then 5 min) for individual C75-treated cells. Each cell is a different colour.

Since C75 destabilizes spindle microtubules, we compared spindle phenotypes with those caused by depletion of CKAP5/ch-TOG, a microtubule polymerase that balances the activity of MCAK, a microtubule depolymerase^13–15,39^. HeLa cells treated with ch-TOG siRNAs had an increase in the proportion of bipolar spindles with misaligned chromosomes (51 vs. 17% for control) and multipolar spindles (42 vs. 8% for control; Fig. 6A). As shown in Fig. 3C, we saw an increase in these phenotypes with increasing amounts of C75. Given these similarities, we determined if C75 impacts ch-TOG localization. Metaphase HeLa cells co-expressing ch-TOG shRNA for endogenous knockdown and RNAi-resistant ch-TOG:GFP were imaged immediately after treatment with 300 nM C75. In control cells, ch-TOG localized to the spindle poles and microtubules, which decreased during anaphase, and was no longer detectable in telophase (13/13; Fig. 6B). Treatment with 300 nM C75 caused ch-TOG to increase at the spindle poles, then dissipate after ~ 50 min (10/10; Fig. 6B). Measuring the change in maximum intensity of ch-TOG at spindle poles within individual cells revealed differences in the signal at one pole relative to the other. While there was some variability in control cells, ch-TOG continued to reciprocally increase or decrease at either pole for a longer time in C75-treated cells (Fig. 6C). We then measured the proportional difference in ch-TOG intensity between the two poles in multiple C75-treated cells where each bar corresponds to a single time point (Fig. 6D). For most cells, ch-TOG alternated from one pole to the other (bars extending to right or left of the net 0 line). This data suggests that C75 treatment causes changes in ch-TOG localization where it first accumulates at the spindle poles, but then unevenly oscillates between the poles as it dissipates. These changes in ch-TOG localization likely prevent it from properly controlling microtubule polymerization.

### C75 and paclitaxel enhance spindle phenotypes at a cellular level

**Figure 7:**
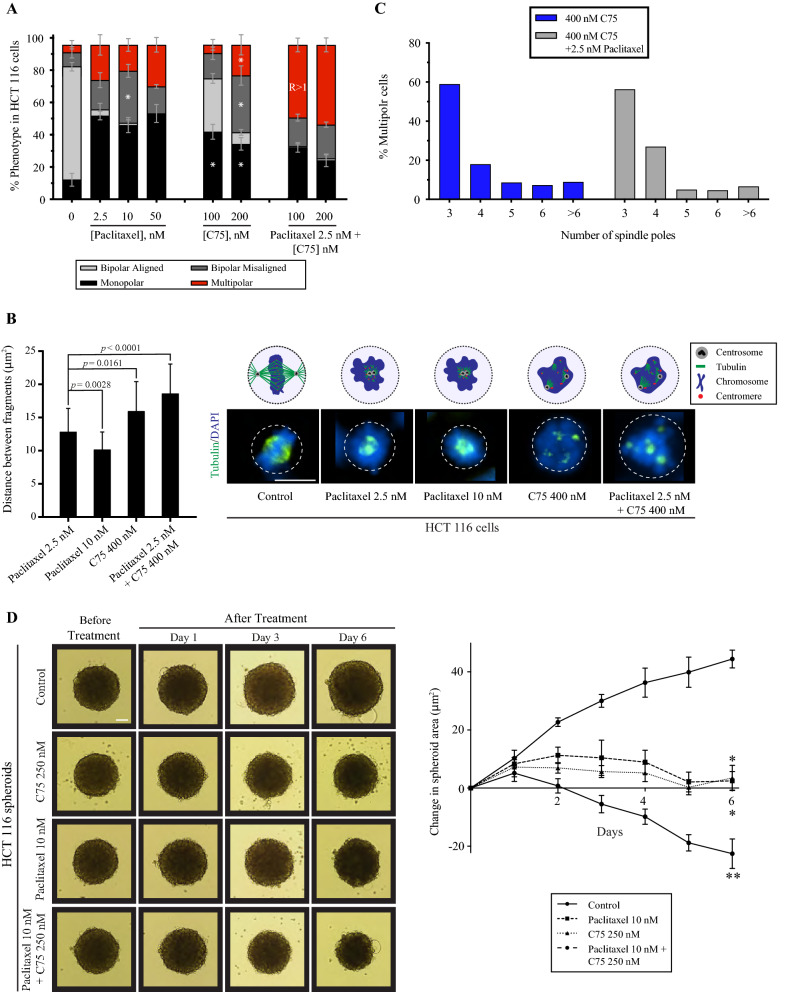
Combining C75 and paclitaxel cause enhanced phenotypes in HCT 116 cells. (**A**) Bar graphs show the proportion of HCT 116 cells with bipolar aligned (light grey), bipolar misaligned (dark grey), multipolar (red) or monopolar (black) spindles 7 h after treatment with control (DMSO), varying concentrations of paclitaxel or C75, or both as indicated. The bars show standard deviation. The asterisks indicate *p* < 0.05 determined by multiple t tests for C75-treated vs. control cells. The combination treatments were compared to predicted ratios as indicated. (**B)** A bar graph shows the distance between spindle poles/fragments measured in HCT 116 cells after treatment with paclitaxel or C75 or both as indicated. Also shown are immunofluorescence images of HCT 116 cells co-stained for DAPI (chromatin; blue) and tubulin (microtubules; green) for the different treatments. The scale bar is 10 µm, and the *p* values are indicated as determined by the student’s t test. (**C)** A bar graph shows the percentage of multipolar HCT 116 cells from B) with different numbers of spindle poles after treatment with 400 nM C75 or 400 nM C75 (blue) and 2.5 nM paclitaxel (light grey). (**D)** Brightfield images show HCT 116 spheroids over 6 days before and after treatment with control (DMSO; n = 5), 250 nM C75 (n = 5), 10 nM paclitaxel (n = 5) or both (n = 5). The scale bar is 0.1 mm. A line graph to the right shows the change in spheroid area (µm^2^) over time. Bars on all graphs show standard deviation. The asterisks are *p* < 0.05 as determined by the student’s t test.

Next, we assessed the effect of combining C75 with paclitaxel, a microtubule-stabilizing drug, in HCT 116 cells. HCT 116 cells were treated with paclitaxel, C75 or both for 7 h, then fixed and co-stained for chromatin (DAPI; blue) and microtubules (green). Paclitaxel caused a significant increase in the proportion of HCT 116 cells with monopolar, bipolar misaligned and/or multipolar spindles (Fig. 7A). The majority of phenotypes did not change between 2.5 and 50 nM of paclitaxel, although 10 nM paclitaxel caused a significant increase in bipolar misaligned spindles (Fig. 7A). As before, increasing concentrations of C75 caused a significant increase in the proportion of cells with spindle phenotypes (Fig. 7A). Combining 2.5 nM paclitaxel with 100 or 200 nM C75 caused an increase in the proportion of cells with multipolar spindles, especially at lower concentrations. To quantify this effect, we calculated the ratio (R) for the observed proportion of cells with multipolar spindles compared to those predicted additively. While R > 1 for 2.5 nM paclitaxel with 100 nM C75 (2.17 ± 0.73), it was close to 1 for 200 (1.45 ± 0.47) and 300 nM (1.19 ± 0.2; Fig. 7A). Thus, the multipolar phenotype caused by C75 was enhanced by paclitaxel.

We noticed that spindle poles were closer together in paclitaxel-treated cells compared to C75. We quantified this by measuring the distance between spindle poles in the different treatments (Fig. 7B). While increasing paclitaxel from 2.5 nM to 10 nM caused the poles to move closer together, 400 nM C75 caused them to move significantly further apart on its own or in combination with paclitaxel (Fig. 7B). This data suggests that C75 and paclitaxel independently affect microtubules, and C75 causes multipolar phenotypes regardless of microtubule stability. In support of this, the number of spindle poles was similar in cells treated with 400 nM C75 alone or in combination with 2.5 nM paclitaxel (Fig. 7C).

Next, we tested the effects of combining C75 and paclitaxel on spheroid growth. Multicellular tumour spheroids are formed by cancer cells grown in 3D. Due to their increased complexity, spheroids better predict the efficacy of drugs in vivo compared to cells grown as monolayers^46,47^. For example, spheroids contain distinct populations of cells that are either quiescent or proliferating. We found that adding 250 nM of C75 or 10 nM of paclitaxel prevented HCT 116 spheroids from growing 6 days after treatment, unlike control spheroids treated with DMSO (Fig. 7D). Combining 250 nM of C75 with 10 nM of paclitaxel caused a significant decrease in spheroid size compared to each compound on their own (Fig. 7D). Thus, a potential application for C75 could be in combination therapies with Taxol to lower doses required to treat cancers.

## Discussion

Here we describe how C75, a microtubule-destabilizing compound, works in cells. C75 is a derivative of a family of thieonoisoquinoline compounds with several groups amenable to modification^43,44^. We previously found that C75 prevents microtubule polymerization and competes with colchicine for tubulin-binding in vitro^44^. Here, we show that C75 caused a range in toxicity depending on the cell type, with stronger effects on HeLa (cervical cancer), A549 (lung cancer) and HCT 116 (colorectal cancer) vs. HFF-1 (foreskin fibroblast) cells. Increasing concentrations of C75 caused cells to accumulate in mitosis, where cells arrested due to aberrant spindles (Figs. 1, 2). While the spindle phenotypes caused by longer treatments of C75 were similar to those caused by colchicine, C75 and colchicine synergized at sub-lethal concentrations, suggesting that they have different effects on microtubules in cells (Fig. 3). Indeed, we found that they caused different spindle phenotypes depending on the cell cycle stage (Figs S3, 4, 5), likely reflecting differences in binding affinity and/or accessibility to microtubules. In addition, microtubules regrew in the presence of C75. To our knowledge, this is not typical for microtubule-destabilizing drugs and we did not observe microtubule recovery with different concentrations of colchicine (e.g. 50 or 300 nM; Figs. 4, 5).

The similarity in spindle phenotypes caused by C75 and ch-TOG depletion is striking^13,14,16^ (Fig. 6). Many MAPs work together to form functional, bipolar spindles^48^. The loss of ch-TOG causes an imbalance in enzymatic activities, including the microtubule depolymerase MCAK. MCAK destabilizes microtubule ends to correct kinetochore attachments, which is controlled by Aurora B kinase, and focuses the asters into spindle poles via Aurora A kinase^11,12,49,50^. ch-TOG-depleted cells have phenotypes consistent with these roles including disorganized spindle poles and misaligned sister chromatids^13^. We observed the same phenotypes in C75-treated cells. Based on this we speculate that: (1) C75 could destabilize the minus ends of microtubules, but after microtubule collapse, its high off-rate permits new polymers to form due to local increases in the critical concentration of free tubulin, (2) C75 disrupts microtubules required for the localization of microtubule regulators, such as ch-TOG, when they are needed to form bipolar spindles^10,16^, and/or (3) changes in accessibility to C75 could arise due to competition with factors that nucleate or polymerize microtubules. The accumulation and oscillation of ch-TOG between spindle poles in C75-treated cells could contribute to changes in microtubule dynamics. For example, ch-TOG requires binding to both microtubule polymers and tubulin dimers to polymerize microtubules, and the loss of microtubules could prevent this function, favoring other activities from other enzymes.

Vinblastine similarly has complex effects on microtubules, which could contribute to its success as an anti-cancer drug. In vitro, vinblastine causes depolymerization at minus ends, and prevents growth at the plus ends^31^. In cells, vinblastine can alter centrioles and/or reduce microtubule dynamics to cause multipolar spindles^32,33^. We propose that C75 shares some similarities with vinblastine with accessibility and/or binding kinetics that favors microtubule depolymerization at the minus ends during mitosis. Vinblastine is one of the few microtubule-destabilizing drugs that is used as an anti-cancer therapy, and its ability to cause spindle phenotypes at low concentrations without disrupting the microtubule polymer mass could minimize side-effects^51^. Although C75 has a high IC_50_ value by comparison (e.g. 30–100-fold higher), the unique effect on microtubules and high selectivity for metaphase cells could make it an interesting compound to consider for in vivo use. Importantly, we are making new derivatives with improved IC_50_ values and solubility that could replace C75 as a lead.

The multipolar spindle phenotype caused by C75 suggests that it affects centrosomes. Centrosome integrity relies on the TACC3-clathrin-ch-TOG complex, and it is interesting that C75 causes loss of integrity prior to the imbalanced recovery of tubulin and ch-TOG at spindle poles^40^. Many microtubule-targeting drugs cause multipolar spindles, but this typically occurs after a long time, or as we showed for colchicine, upon mitotic entry^32,52–54^. Cancer cells with high aneuploidy and amplified centrosomes rely on clustering mechanisms to form bipolar spindles^38,55–57^. Thus, C75 may be effective for the treatment of cancers with high centrosome numbers^55^. Indeed, the proportion of cells with multipolar spindles caused by C75 varied among the different cancer cell types, likely reflecting genetic differences impacting centrosome integrity, clustering and/or microtubule dynamics. Interestingly, low concentrations of C75 caused an increase in the proportion of monopolar spindles in HCT 116 cells. Aurora A kinase and ch-TOG are over-expressed in HCT 116 cells causing increased microtubule assembly^14,36–42^. Given its potentially high off-rate, low concentrations of C75 could increase critical concentrations of free tubulin to further stabilize microtubules and block centrosome separation. However, higher concentrations of C75 caused the formation of multipolar spindles, including cells that had also been treated with paclitaxel, which stabilizes microtubules (Figs. 3, 7). We also found that combining C75 and paclitaxel more effectively regressed multicellular tumour spheroids compared to each on their own (Fig. 7). One of the compounds could have improved accessibility due to changes in the microtubule polymer caused by the other compound, and spindles are less likely to recover if subthreshold doses can cause additive effects. Our data supports the exploration of C75’s anti-cancer potential, including predicting cell types that could respond to C75 with high efficacy.

## Experimental procedures

### Cell culture and drug treatments

HeLa and HFF-1 cells obtained from the American Type Culture Collection (ATCC) were grown in DMEM (Wisent), while A549 cells were grown in F12K media (Wisent) and HCT 116 (p53-/-) cells were grown in McCoy’s media (Wisent) in humidified incubators at 37 °C with 5% CO_2_. All media were supplemented with 10% fetal bovine serum (FBS; Thermo Scientific), 2 mM l-glutamine (Wisent), 100 U penicillin (Wisent), and 0.1 mg/mL streptomycin (Wisent).

C87 and C75 were stored at − 20 °C as 1 mM stocks in dimethyl sulfoxide (DMSO). Working stocks of 100 μM C75 or C87 were made in DMSO:H_2_O (9:1). Colchicine (Sigma) was dissolved in ethanol as a 1 M stock and diluted to 10 μM before use. Nocodazole was dissolved in DMSO as a 1 mg/mL stock (Sigma). Paclitaxel (Bioshop) was dissolved in DMSO as a 10 mM stock and diluted to a range of smaller concentrations before use. Cells grown as monolayer cultures or as spheroids were treated with C75, C87, colchicine, or paclitaxel as indicated, and the final concentrations of DMSO or ethanol were kept below 0.5%.

### Tubulin binding assays

Tubulin binding was assessed using a fluorescence quenching assay^58^. This assay was performed with 1.5 uM of purified tubulin taken from a flash frozen 10 mg/mL stock (Cytoskeleton Inc.) thawed on ice and diluted in 600 μL of 25 mM PIPES pH 6.9. Tubulin was placed in a Q fluorometer cell, Z = 20 (Varian), and incubated with DMSO, or 5 μM of C87, C75 or nocodazole at 25 °C for 20 min. Measurements were taken using a fluorescence spectrophotometer Cary Eclipse (Varian). The sample was excited at 295 nm and the fluorescence intensity was measured over a range of emission spectra from 300 to 500 nm. Scans were repeated 10 times through computer averaging of transients (CAT) application which averages each individual scan. All values were exported as excel files and graphs were generated in Graphpad Prism v.9.2.0. The experiment was replicated three times.

### Viability assays

Assays were performed to determine the viability of cells after treatment with C75 and/or colchicine. The concentration of colchicine or C75 used in the combination experiments was the highest concentration that caused little to no toxicity based on dose–response curves. HFF-1, HeLa, A549 and HCT 116 cells were plated with 4,000 cells/well in 96-well dishes and left overnight to adhere. Drug dilutions were prepared and added to the cells using an acoustic liquid handler (LabCyte ECHO 550). After 3 population doubling times, cells were assessed for viability using the WST-8 cell proliferation assay kit (Cayman Chemicals). Absorbance readings at 450 nm were collected using the TECAN 200 PRO plate reader. Values for each replicate were adjusted to the controls and plotted using Graphpad Prism v.9.2.0 to generate graphs and IC_50_ values. All assays were performed in triplicate for each treatment. For the combination assays, C75 or colchicine were repeated alongside the combination treatments to ensure accurate comparison.

### Flow cytometry

Flow cytometry was used to measure changes in the proportion of cells in different stages of the cell cycle after C75-treatment. HeLa, HCT 116 and A549 cells were grown to 80% confluency and treated with a range of C75 concentrations for 8 h. Cells were harvested in falcon tubes, then fixed with 70% cold ethanol and washed with cold phosphate buffered saline (PBS; Wisent). Cells were permeabilized and stained for 15 min at 37 °C with a solution containing 500 μg/mL PI (Sigma) in PBS with 0.1% (v/v) Triton X-100 and DNAse-free RNAse A (Sigma). Cells were protected from light and measured for PI intensity using the BD LSRFortessa flow cytometer with excitation at 561 nm and detection at 600 nm (LP) using the D-BP filter. Each treatment was done in triplicate with 20,000 cells per sample analyzed using FCS 7 Cytometry (https://denovosoftware.com/). Data was exported and plotted using GraphPad Prism v.9.2.0 to make bar graphs.

### Immunofluorescence staining

Immunofluorescence was performed to monitor mitotic spindle phenotypes. Cultured cells were plated on coverslips at a confluency of 40–50% and left overnight to adhere. Cells were fixed using freshly prepared ice-cold 10% w/v cold trichloroacetic acid (TCA) for 14 min at 4 °C. Cells were washed with PBST (0.3% Triton X-100) and kept at 4 °C prior to staining. After blocking, fixed cells were immunostained for microtubules using 1:400 mouse anti-α-tubulin antibodies (DM1A; Sigma) or centrosomes using 1:400 mouse anti-γ-tubulin antibodies (Santa Cruz Biotechnology) or mouse anti-Centrin 2 (clone 20H5; Sigma), and centromeres using 1:500 human anti-centromere antibodies (ACA; Sigma) for 2 h at room temperature. After washing, anti-mouse Alexa 488 and anti-human Alexa 647 (Invitrogen) secondary antibodies were used at a 1:500 dilutions for 2 h at room temperature. After washing, 4′,6-Diamidino-2′-phenylindole dihydrochloride (DAPI; Sigma) was added for 5 min. Cells were then washed with PBST, followed by a wash with 0.1 M Tris pH 9, then a drop of mounting media (0.5% propyl gallate in 50% glycerol) was added to the coverslip, which was mounted onto a slide and sealed.

### Microscopy

Fixed slides were imaged using the Nikon-TIE inverted epifluorescence microscope with Lambda XL LED light sources, using the 60x/1.4 oil objective, a Piezo Z stage (ASI), a Photometrics Evolve 512 EMCCD camera and NIS Elements (Nikon). Exposures were determined by control cells, and the same settings were used in the treatment conditions. Images were acquired as 1 μm Z-stacks and exported as TIFFs, which were opened as maximum intensity Z-stack projections in Image J v.2.0.0 (NIH; https://imagej.nih.gov/ij/index.html). Merged colour images were converted into 8-bit images and imported into Adobe Illustrator v.25 to make figures.

For live imaging, HeLa cells were plated to 50–60% confluency on 25 mm round coverslips (No. 1.5; Neuvitro). Cells were treated with 75 nM Hoescht 33,342 and 200 nM SiR-tubulin (Cytoskeleton Inc.) for 90 min prior to imaging. Depending on the experiment, HeLa cells were transfected with a plasmid that expresses RNAi-resistant ch-TOG:GFP plus a hairpin RNA (sh ch-TOG) to knockdown endogenous protein and minimize overexpression (Addgene ID# 69,113). HeLa cells stably expressing GFP:tubulin and mCherry:tubulin were previously generated^59^. Coverslips were transferred to a 35 mm chamlide magnetic chamber (Quorum) and kept at 37 °C with 5% CO_2_ using an INU-TiZ-F1 chamber (MadCityLabs). Images were acquired at 1 or 5-min intervals using the 60x/1.4 oil objective on the Nikon Livescan sweptfield confocal microscope with an Andor iXon X3 EMCCD camera and NIS Elements (Nikon). The 405 nm, 480 nm and 640 nm lasers were used to image Hoescht, GFP and sir-Tubulin, respectively, with a quad filter and exposures set to controls. Z-stacks of 1 μm were collected using a NI-DAQ piezo Z stage (National Instruments). Images were used to make figures as described above.

HeLa cells that stably express GFP:tubulin or mCherry:tubulin were plated onto coverslips and treated with 50 nM colchicine or 300 nM C75 for 2 h and transferred to a 35 mm chamlide magnetic chamber (Quorum). The cells were transferred to 37 °C with 5% CO_2_ using an INU-TiZ-F1 chamber (MadCityLabs) and random fields of view were chosen on the cover slip and imaged for > 4 h using 60x/1.4 oil objective on the Nikon Livescan sweptfield confocal microscope with an Andor iXon X3 EMCCD camera and NIS Elements (Nikon). The 480 nm laser was used to image GFP:tubulin with a quad filter and exposures set to controls. Z-stacks of 1 μm were collected using a NI-DAQ piezo Z stage (National Instruments). Images were used to make figures as described above.

To analyze the recovery of microtubules after cold-treatment in cells, HeLa cells were plated onto coverslips and transferred to a 35 mm chamlide magnetic chamber (Quorum), which was placed in an ice-cold water bath to cause spindle collapse. After 30 min, the cells were transferred to 37 °C with 5% CO_2_ using an INU-TiZ-F1 chamber (MadCityLabs) and C75 or colchicine was added after acquisition of the first timepoint. Images were acquired using the 60x/1.4 oil objective on the Nikon Livescan sweptfield confocal microscope with an Andor iXon X3 EMCCD camera and NIS Elements (Nikon). The 405 nm and 480 nm lasers were used to image Hoescht 33,342, GFP:tubulin respectively, with a quad filter and exposures set to controls. Z-stacks of 1 μm were collected using a NI-DAQ piezo Z stage (National Instruments). Images were used to make figures as described above.

### Multicellular tumour spheroids

HCT 116 spheroids were grown on agarose. In a 96-well dish, 1,000 HCT 116 cells were added to wells coated with 1.5% agarose. After spheroids grew to an appropriate size and appearance (~ 5 days), they were transferred to agarose-coated wells in a 24-well dish in 1 mL of media. After the addition of DMSO, C75, paclitaxel or both C75 and paclitaxel in combination, images were collected every 24 h for 6 days using a Nikon-TIE inverted microscope with the 4x/0.2 objective and the 1.5 magnification changer using the DS-Ri2 ultra high-resolution colour camera (Nikon) and NIS Elements (Nikon).

### Analysis

For the analysis of spindle phenotypes, 1 mm z-stack projections of individual cells were used. To calculate the proportion of mitotic cells in Fig. 2A, we counted the following number of rounded HeLa cells: 0 nM n = 568, 100 nM n = 847, 200 nM n = 266, 300 nM n = 105, 400 nM n = 96, 500 nM n = 75), A549 cells: 0 nM n = 461, 100 nM n = 519, 200 nM n = 181, 300 nM n = 154, 400 nM n = 150, 500 nM n = 253), and HCT 116 cells: 0 nM n = 813, 100 nM n = 742, 200 nM n = 297, 300 nM n = 296, 400 nM n = 314, 500 nM n = 301 (N = 4 experimental replicates). To calculate the proportion of cells with different mitotic spindle phenotypes in Fig. 2B,C, the following number of HFF-1 cells were counted after treatment with DMSO n = 85, 300 nM C87 n = 99 and 300 nM C75 n = 84, HeLa cells after treatment with DMSO n = 152, 300 nM C87 n = 147 and 300 nM C75 n = 332, A549 cells with DMSO n = 87, 300 nM C87 n = 130 and 300 nM C75 n = 204, and HCT 116 cells with DMSO n = 131, 300 nM C87 n = 128 and 300 nM C75 n = 245 (N = 3 experimental replicates). To determine the number of centrin-2-positive foci in HeLa cells (Fig. 2D), an average of 25 cells were counted for the control (DMSO-treated) and 30 cells after treatment with 300 nM C75 (N = 3 experimental replicates). To calculate changes in the proportion of mitotic spindle phenotypes in HeLa cells after treatment with C75 and/or colchicine (Fig. 3B,C), we counted the following numbers of cells: DMSO n = 128, 20 nM colchicine n = 186, 50 nM colchicine n = 215, 100 nM colchicine n = 223, 100 nM C75 n = 127, 200 nM C75 n = 134, 300 nM C75 n = 370, 400 nM C75 n = 219, 500 nM C75 n = 200, 100 nM C75 + 20 nM colchicine n = 223, 200 nM C75 + 20 nM colchicine n = 273, 300 nM C75 + 20 nM colchicine n = 321, 400 nM C75 + 20 nM colchicine n = 303 and 500 nM C75 + 20 nM colchicine n = 277 (N = 3 experimental replicates). To count the proportion of cells with multipolar or bipolar spindles in HeLa cells after release from C75 or colchicine (Fig. 4B), we counted the following number of cells: DMSO n = 61, 500 nM C75 n = 50 and 500 nM colchicine n = 64 (N = 3 experimental replicates). For Fig. 6A, we counted 534 ch-TOG RNAi cells and 81 DMSO cells (N = 3 experimental replicates). To calculate changes in the proportion of mitotic spindle phenotypes in HCT 116 cells after treatment with C75 and/or paclitaxel (Fig. 7A), we counted the following number of cells: DMSO n = 116, 2.5 nM paclitaxel n = 99, 10 nM paclitaxel n = 91, 50 nM paclitaxel n = 79, 100 nM paclitaxel n = 75, 100 nM C75 n = 52, 200 nM C75 n = 53, 2.5 nM paclitaxel + 100 nM C75 n = 112, 2.5 nM paclitaxel + 200 nM C75 n = 72 (N = 3 experimental replicates). To measure the distance between fragmented spindle poles in Fig. 7B, we counted the following number of cells: 2.5 nM paclitaxel n = 21, 10 nM paclitaxel n = 21, 2.5 nM paclitaxel + 400 nM C75 n = 31, 2.5 nM paclitaxel + 400 nM C75 n = 22 (N = 3 experimental replicates).

To determine synergy for the viability of cells treated with C75 and colchicine in combination, CompuSyn (https://www.combosyn.com/index.html) was used (Figs. 3A, S2A). We used the non-constant ratio method of analysis since the concentration used for the combination studies was selected based on the highest concentration without lethality. The analysis indicated what combination of drug concentrations yielded an antagonistic, synergistic or additive effect based on the well-documented combination index (CI) described by the Chou-Talalay method^45^.

To determine the effect of C75 on spindle poles, images of HeLa cells stably expressing GFP:Tubulin and/or ch-TOG:GFP; sh ch-TOG were collected and used to measure changes in spindle volume and maximum intensity. To do this, z-stacks of images collected by sweptfield confocal microscopy were deconvolved using Autoquant and then reconstructed into 3D cells by rendering in Imaris v.9.7.2 (Bitplane). Thresholding was used to identify the spindle poles and render them as distinct objects using the spot analyzer function. The spherical objects were tracked and quantified over time for changes in their volume and intensity. The values were exported and organized in csv format using a macro in Python v.3.0 (https://www.python.org/download/releases/3.0/) to extract the volume and maximum intensity of each pole. The csv files were then imported into GraphPad Prism v.9.2.0 to build graphical representations, including heat maps, bar graphs and distribution plots. Statistical analyses were also done in GraphPad Prism v.9.2.0, to determine the significance between slope distributions using the Welch’s two-tailed t test with a 99% confidence interval (*p* < 0.0001). Peaks corresponding to changes in spindle pole volume was determined for each pole using the built-in function “findpeaks” in MatLab R2017b (coding in Spyder v.4.0.1; https://www.spyder-ide.org). The parameters were set to identify any peaks that corresponded to a change in volume with an amplitude >  = 20%. Identified peaks were then tabulated in Excel (Microsoft) and imported to GraphPad Prism v.9.2.0 for graphical representation.

The distances between spindle fragments in Fig. 7B were analyzed using a macro written for Image J v.2.0.0 (NIH; https://imagej.nih.gov/ij/index.html). Image files were exported as TIFFs and opened as Z-stack projections of maximum intensity and converted to a binary mask. Using the ROI manager, each cell was first identified using DAPI, then the tubulin fragments were identified. Each fragment correlated with a point of maximum intensity, and the coordinates for that point were recorded. The distance between two points was measured using the coordinates of each point, and triangulating the distance based on Pythagorean theorem: A2 + B2 = C2, C being the distance between 2 given points.

To measure changes in spheroid area in Fig. 7C, images were exported as TIFFs and analyzed using a macro written for Image J v.2.0.0 (NIH; https://imagej.nih.gov/ij/index.html). Images were subjected to "IsoData" thresholding and 10 iterations of the “close” function to fill in pixel size gaps. Spheroids were identified using the “analysis of particles” command and the area was measured from the radius generated by the software.

## Supplementary Information

Supplementary Information.

## Data Availability

The data generated during and/or analysed during the current study are available from the corresponding author on reasonable request.

## References

[1] Musacchio A (2015). The molecular biology of spindle assembly checkpoint signaling dynamics. Curr. Biol..

[2] Shi J, Mitchison TJ (2017). Cell death response to anti-mitotic drug treatment in cell culture, mouse tumor model and the clinic. Endocrine-Relat. Cancer.

[3] Oakley BR, Paolillo V, Zheng Y (2015). γ-Tubulin complexes in microtubule nucleation and beyond. Mol. Biol. Cell.

[4] Martin M, Akhmanova A (2018). Coming into focus: Mechanisms of microtubule minus-end organization. Trends Cell Biol..

[5] Brouhard GJ, Rice LM (2018). Microtubule dynamics: An interplay of biochemistry and mechanics. Nat. Rev. Mol. Cell Biol..

[6] Wordeman L (2019). GTP-tubulin loves microtubule plus ends but marries the minus ends. J. Cell Biol..

[7] Akhmanova A, Steinmetz MO (2015). Control of microtubule organization and dynamics: Two ends in the limelight. Nat. Rev. Mol. Cell Biol..

[8] Gigant B (2005). Structural basis for the regulation of tubulin by vinblastine. Nature.

[9] Goodson HV, Jonasson EM (2018). Microtubules and microtubule-associated proteins. Cold Spring Harbor Persp. Biol..

[10] Widlund PO (2011). XMAP215 polymerase activity is built by combining multiple tubulin-binding TOG domains and a basic lattice-binding region. Proc. Natl. Acad. Sci..

[11] Zhang X, Ems-McClung SC, Walczak CE (2008). Aurora A phosphorylates MCAK to control Ran-dependent spindle bipolarity. Mol. Biol. Cell.

[12] Walczak CE, Gayek S, Ohi R (2013). Microtubule-depolymerizing kinesins. Annu. Rev. Cell Dev. Biol..

[13] Gergely F, Draviam VM, Raff JW (2003). The ch-TOG/XMAP215 protein is essential for spindle pole organization in human somatic cells. Genes Dev..

[14] Barr AR, Bakal C (2015). A sensitised RNAi screen reveals a ch-TOG genetic interaction network required for spindle assembly. Sci. Rep..

[15] Barr AR, Gergely F (2008). MCAK-independent functions of ch-Tog/XMAP215 in microtubule plus-end dynamics. Mol. Cell. Biol..

[16] Brouhard GJ (2008). XMAP215 is a processive microtubule polymerase. Cell.

[17] Dominguez-Brauer C (2015). Targeting mitosis in cancer: Emerging strategies. Mol. Cell.

[18] Hanahan D, Weinberg RA (2011). Hallmarks of cancer: The next generation. Cell.

[19] Weaver BA (2014). How taxol/paclitaxel kills cancer cells. Mol. Biol. Cell.

[20] Kumar N (1981). Taxol-induced polymerization of purified tubulin. Mechanism of action. J. Biol. Chem..

[21] Parness J, Horwitz S (1981). Taxol binds to polymerized tubulin in vitro. J. Cell Biol..

[22] Zasadil LM (2014). Cytotoxicity of paclitaxel in breast cancer is due to chromosome missegregation on multipolar spindles. Sci. Transl. Med..

[23] Kavallaris M (2010). Microtubules and resistance to tubulin-binding agents. Nat. Rev. Cancer.

[24] Fitzgerald TJ (1976). Molecular features of colchicine associated with antimitotic activity and inhibition of tubulin polymerization. Biochem. Pharmacol..

[25] Kumar A, Sharma PR, Mondhe DM (2017). Potential anticancer role of colchicine-based derivatives. Anticancer Drugs.

[26] Massarotti A, Coluccia A, Silvestri R, Sorba G, Brancale A (2012). The tubulin colchicine domain: A molecular modeling perspective. ChemMedChem.

[27] Wang Y (2016). Structures of a diverse set of colchicine binding site inhibitors in complex with tubulin provide a rationale for drug discovery. FEBS J..

[28] Field JJ, Kanakkanthara A, Miller JH (2014). Microtubule-targeting agents are clinically successful due to both mitotic and interphase impairment of microtubule function. Bioorg. Med. Chem..

[29] Jordan M, Kamath K (2007). How do microtubule-targeted drugs work? An overview. Curr. Cancer Drug Targets.

[30] Martino E (2018). Vinca alkaloids and analogues as anti-cancer agents: Looking back, peering ahead. Bioorg. Med. Chem. Lett..

[31] Panda D, Miller HP, Islam K, Wilson L (1997). Stabilization of microtubule dynamics by estramustine by binding to a novel site in tubulin: A possible mechanistic basis for its antitumor action. Proc. Natl. Acad. Sci..

[32] Jordan MA, Thrower D, Wilson L (1992). Effects of vinblastine, podophyllotoxin and nocodazole on mitotic spindles: Implications for the role of microtubule dynamics in mitosis. J. Cell Sci..

[33] Wendell KL, Wilson L, Jordan MA (1993). Mitotic block in HeLa cells by vinblastine: Ultrastructural changes in kinetochore-microtubule attachment and in centrosomes. J. Cell Sci..

[34] Brito DA, Rieder CL (2009). The ability to survive mitosis in the presence of microtubule poisons differs significantly between human nontransformed (RPE-1) and cancer (U2OS, HeLa) cells. Cell Motility Cytoskeleton.

[35] Hedrick DG, Stout JR, Walczak CE (2008). Effects of anti-microtubule agents on microtubule organization in cells lacking the kinesin-13 MCAK. Cell Cycle.

[36] Al-Bassam J, Chang F (2011). Regulation of microtubule dynamics by TOG-domain proteins XMAP215/Dis1 and CLASP. Trends Cell Biol..

[37] Byrnes AE, Slep KC (2017). TOG–tubulin binding specificity promotes microtubule dynamics and mitotic spindle formation. J. Cell Biol..

[38] Fielding AB, Lim S, Montgomery K, Dobreva I, Dedhar S (2011). A critical role of integrin-linked kinase, ch-TOG and TACC3 in centrosome clustering in cancer cells. Oncogene.

[39] Holmfeldt P, Stenmark S, Gullberg M (2004). Differential functional interplay of TOGp/XMAP215 and the KinI kinesin MCAK during interphase and mitosis. EMBO J..

[40] Hood FE (2013). Coordination of adjacent domains mediates TACC3–ch-TOG–clathrin assembly and mitotic spindle binding. J. Cell Biol..

[41] Yu N (2016). Isolation of functional tubulin dimers and of tubulin-associated proteins from mammalian cells. Curr. Biol..

[42] Ertych N (2014). Increased microtubule assembly rates influence chromosomal instability in colorectal cancer cells. Nat. Cell Biol..

[43] Chen F, Wong NWY, Forgione P (2014). One-pot tandem palladium-catalyzed decarboxylative cross-coupling and C-H activation route to Thienoisoquinolines. Adv. Synth. Catal..

[44] Liu JT (2021). Design, structure-activity relationship study and biological evaluation of the thieno[3,2-c]isoquinoline scaffold as a potential anti-cancer agent. Bioorg. Med. Chem. Lett..

[45] Chou T-C (2010). Drug combination studies and their synergy quantification using the Chou-Talalay method. Cancer Res..

[46] Friedrich J, Seidel C, Ebner R, Kunz-Schughart LA (2009). Spheroid-based drug screen: Considerations and practical approach. Nat. Prot..

[47] Sutherland RM (1988). Cell and environment interactions in tumor microregions: The multicell spheroid model. Science.

[48] Petry S (2016). Mechanisms of mitotic spindle assembly. Annu. Rev. Biochem..

[49] Ems-McClung SC (2013). Aurora B inhibits MCAK activity through a phosphoconformational switch that reduces microtubule association. Curr. Biol..

[50] Andrews PD (2004). Aurora B regulates MCAK at the mitotic centromere. Dev. Cell.

[51] Hughes J, Rees S, Kalindjian S, Philpott K (2011). Principles of early drug discovery. Br. J. Pharmacol..

[52] Levrier C (2017). 6α-Acetoxyanopterine: A novel structure class of mitotic inhibitor disrupting microtubule dynamics in prostate cancer cells. Mol. Cancer Therapeut..

[53] Foraker AB (2012). Clathrin promotes centrosome integrity in early mitosis through stabilization of centrosomal ch-TOG. J. Cell Biol..

[54] Karki M, Keyhaninejad N, Shuster CB (2017). Precocious centriole disengagement and centrosome fragmentation induced by mitotic delay. Nat. Commun..

[55] Leber B (2010). Proteins required for centrosome clustering in cancer cells. Sci. Transl. Med..

[56] Ogden A, Rida PCG, Aneja R (2012). Let’s huddle to prevent a muddle: Centrosome declustering as an attractive anticancer strategy. Cell Death Differ..

[57] Sabat-Pośpiech D, Fabian-Kolpanowicz K, Prior IA, Coulson JM, Fielding AB (2019). Targeting centrosome amplification, an Achilles’ heel of cancer. Biochem. Soc. Trans..

[58] Rai, A., Surolia, A. & Panda, D. An antitubulin agent BCFMT inhibits proliferation of cancer cells and induces cell death by inhibiting microtubule dynamics. *PLOS ONE* **7**(8), e44311. 10.1371/journal.pone.0044311 (2012). 10.1371/journal.pone.0044311 PMC3432122 22952952

[59] van OostendeTriplet C, JaramilloGarcia M, HajiBik H, Beaudet D, Piekny A (2014). Anillin interacts with microtubules and is part of the astral pathway that defines cortical domains. J. Cell Sci..

