# Supplementary material for: Characterization of a recently synthesized microtubule-targeting compound that disrupts mitotic spindle poles in human cells
Source: Sci Rep. 2021 Dec 8;11:23665. doi: 10.1038/s41598-021-03076-3 (PMC8655040; doi:10.1038/s41598-021-03076-3)

Supplementary Information

Characterization of a recently synthesized microtubule-targeting compound that disrupts mitotic spindle poles in human cells

Dilan Boodhai Jaunky[1*], Kevin Larocque[1*], Mathieu C. Husser[1], Jiang Tian Liu[2], Pat Forgione[2], Alisa Piekny[1^]

1. Department of Biology, Concordia University, Montreal, QC, Canada
2. Department of Chemistry and Biochemistry, Concordia University, Montreal, QC, Canada

*Equal contribution

^Correspondence: Alisa Piekny, PhD, Associate Professor; alisa.piekny@concordia.ca

Keywords: centrosome, colchicine, microtubules, mitosis, spindle

Supplemental Figure 1. C75 causes cells to arrest in G2/M. A) Graphs show the proportion of HeLa, A549 and HCT 116 cells in the different phases of the cell cycle (G1 in green; S in yellow; G2/M in purple) 8 hours after treatment with varying concentrations of C75, as determined by flow cytometry. Cell counts are on the Y-axis and propidium iodide (PI) intensity (levels) is on the X-axis. **B)** DIC images show fields of view of HeLa cells after addition of DMSO (control), 300 nM C75 or 50 nM colchicine over time for 17 hours. White and black arrows point to cells. The scale bar is 10 μm.

Supplemental Figure 2. Combining C75 and colchicine cause enhanced lethality and spindle phenotypes. A) Tables illustrate the percent lethality of HeLa cells treated with colchicine (top left), C75 (top right), or both in combination (bottom two) for different concentrations as indicated. Also included are values for the Combination Index (CI) calculated using CompuSyn software, and whether the values indicate synergistic, antagonistic or additive interactions. Red boxes highlight the lowest concentrations that show synergism between C75 and colchicine. **B)** A bar graph shows the percentage of multipolar HeLa cells from Figure 3C with different numbers of

spindle poles after treatment with 300 nM C75 (black), 500 nM C75 (blue), 50 nM colchicine (dark grey) or 300 nM colchicine plus 20 nM colchicine (light grey).

Supplemental Figure 3. Colchicine causes centrosome fragmentation prior to metaphase. A) Timelapse images show live HeLa cells expressing GFP:tubulin (green) without (control, n=12) or after treatment with 50 nM colchicine (n=74). Times are indicated in minutes. The proportion of cells with bipolar or multipolar spindles are indicated. **B)** Timelapse images show HeLa cells expressing mCherry:tubulin (red) without (control, n=22) or after treatment with 300 nM C75 (n=44). Times are indicated in minutes. The proportion of cells with bipolar, multipolar or monopolar spindles are indicated. The scale bar for cells in A) and B) is 10 μm.

Supplemental Figure 4. Spindle poles recover in the presence of C75. A) A scatter plot shows the distribution of best-fit slope values obtained from plotting changes in the maximum intensity of tubulin at spindle poles over time after treatment with 300 nM colchicine or C75. Statistics were done using two-tailed Welch's t test with a $p<0.0001$. **B)** Heat maps show the change in maximum intensity of tubulin at each spindle pole (%) over time (in minutes) after treatment with 300 nM colchicine (top) or C75 (bottom). Purple indicates high values, while orange indicates low values.

Supplemental Data 1

A

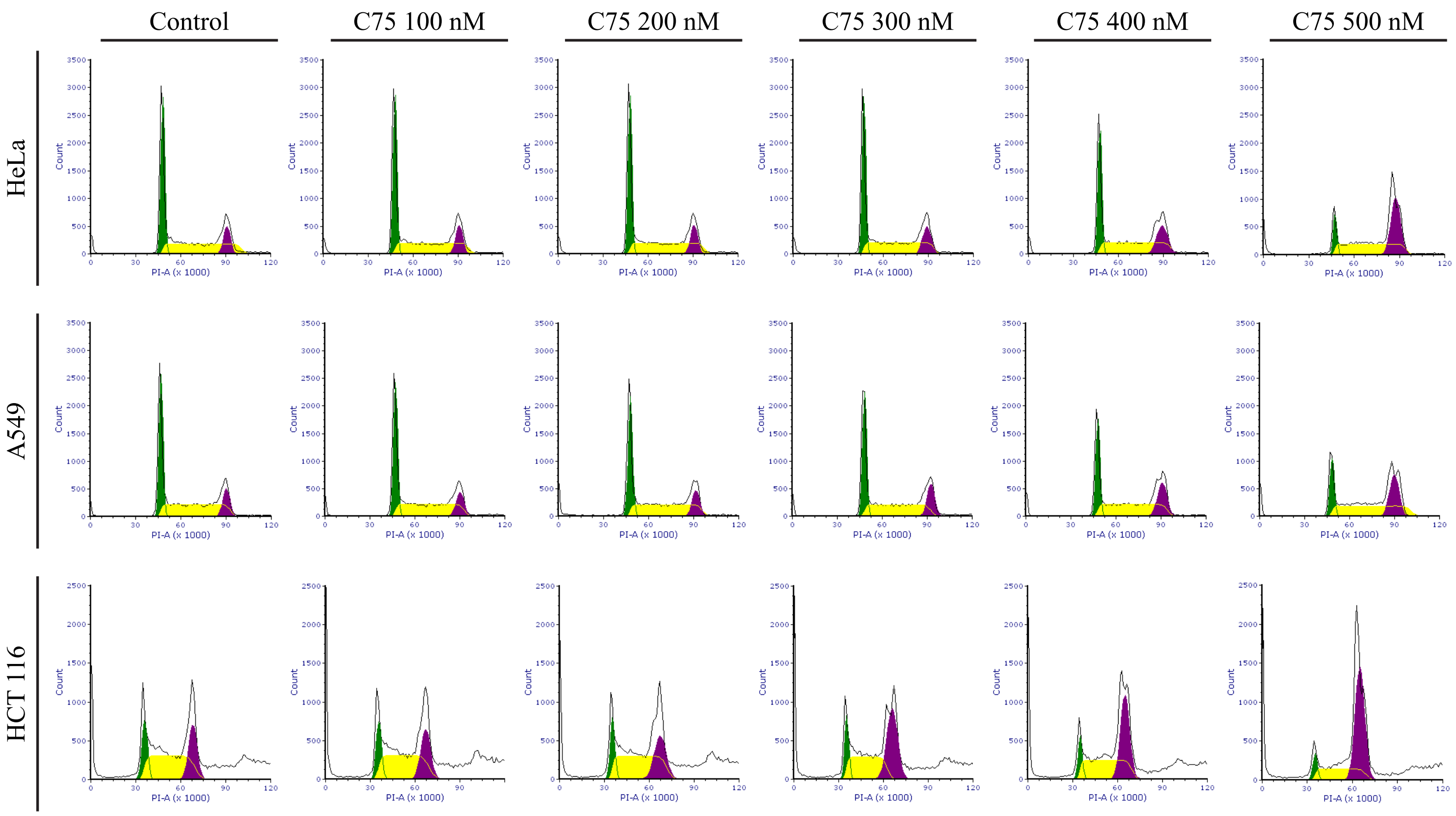

B

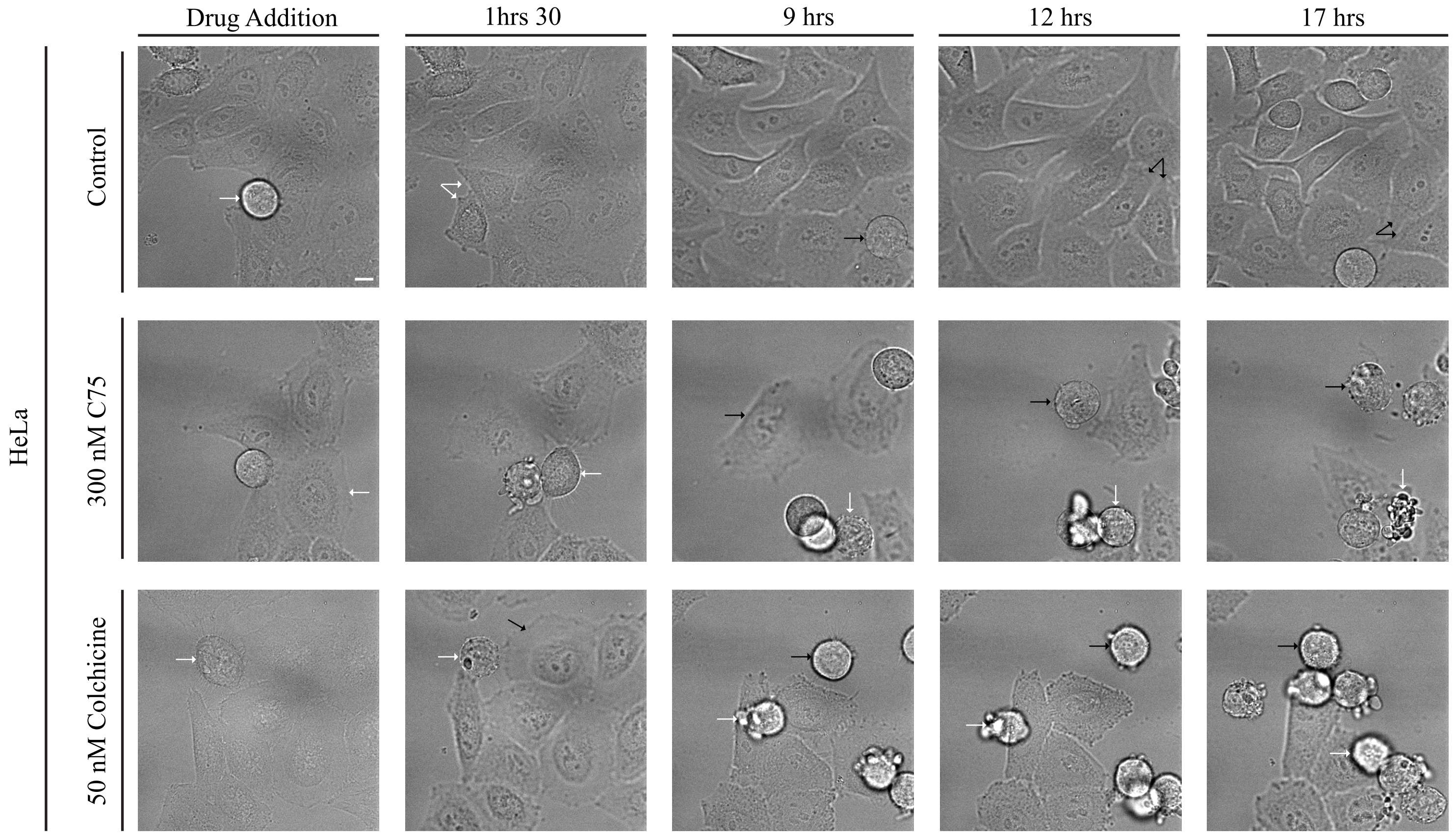

A

[Colchicine], nM	% Lethality
0	0%
1	2%
5	2%
10	58%
20	99%
30	99%
40	99%
50	100%
100	100%
1000	100%

[C75], nM	% Lethality
0	0%
1	6%
10	10%
100	0%
200	9%
300	22%
400	23%
500	97%
700	99%
1000	100%

[Colchicine], nM	[C75], nM	% Lethality	Combination Index (CI)	
0	0	0%	>1000	Antagonistic
3	0	19%	7.4	Antagonistic
3	100	14%	237	Antagonistic
3	300	95%	0.2	Synergistic
3	500	100%	0	-
3	1000	100%	0	-
3	10000	99%	0	-

[C75], nM	[Colchicine], nM	% Lethality	Combination Index (CI)	
0	0	0%	>1000	Antagonistic
250	0	2%	>1000	Antagonistic
250	1	12%	897	Antagonistic
250	5	96%	0.25	Synergistic
250	10	100%	0	-
250	20	100%	0	-
250	30	99%	0	-
250	40	99%	0	-
250	50	100%	0	-
250	100	100%	0	-
250	1000	100%	0	-

B

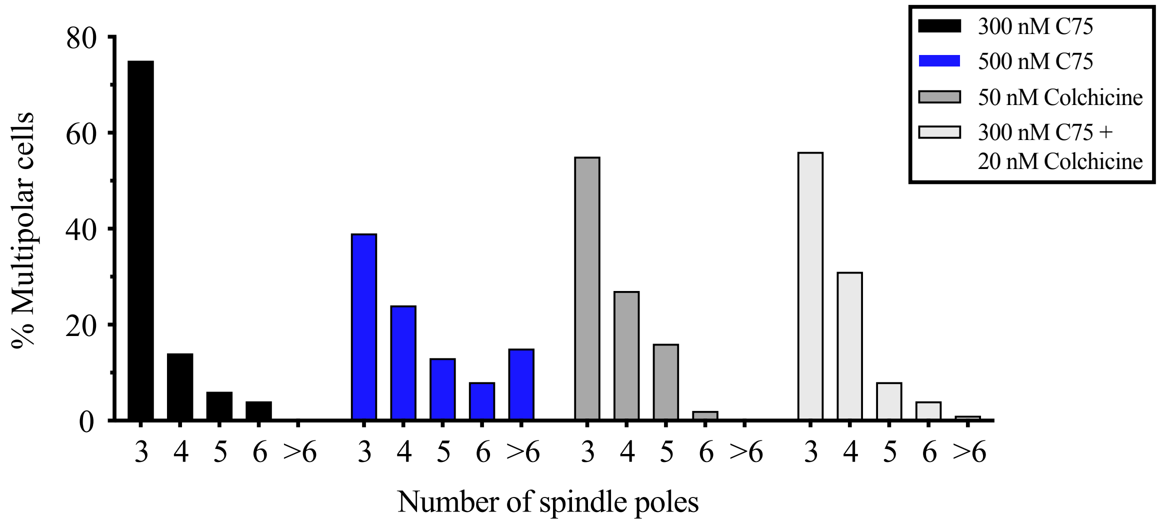

Supplemental Data 3

A

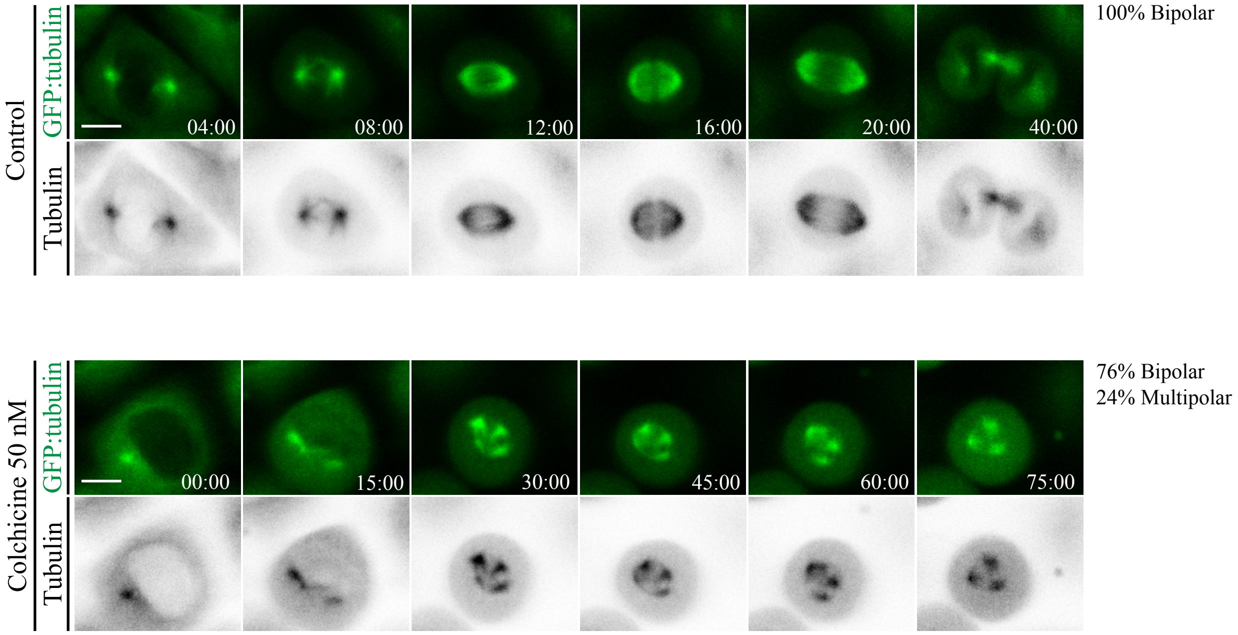

B

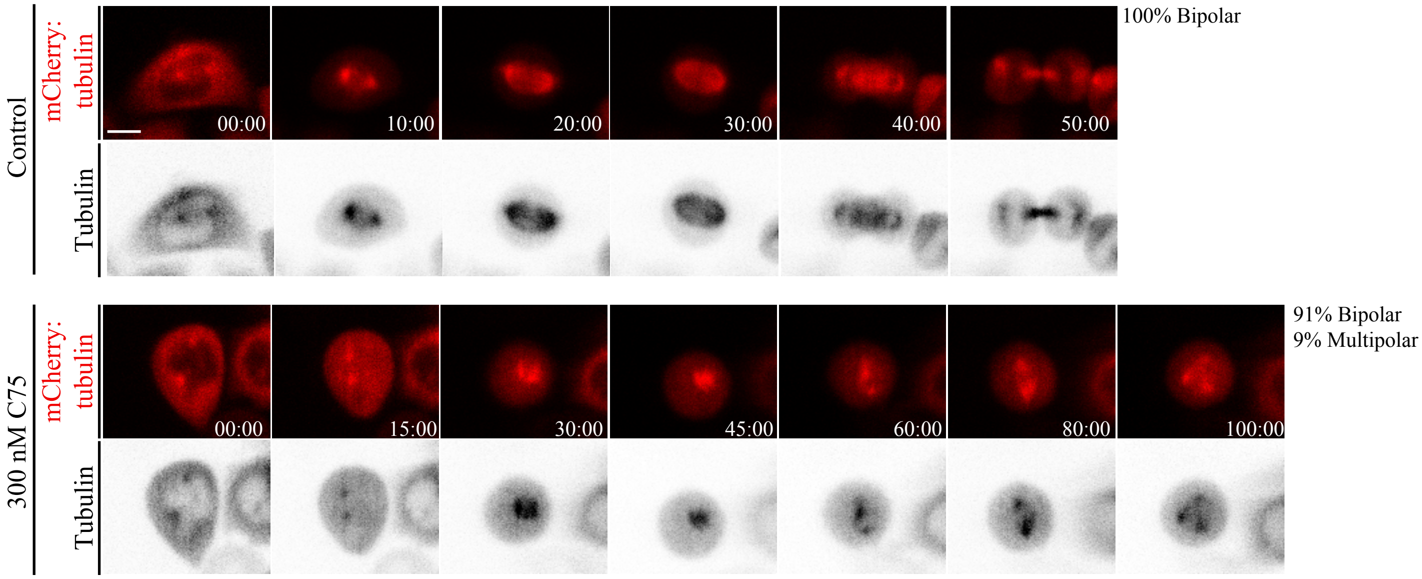

Supplemental Data 4

A

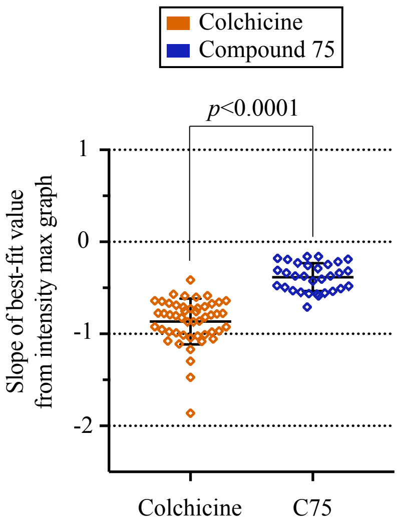

B

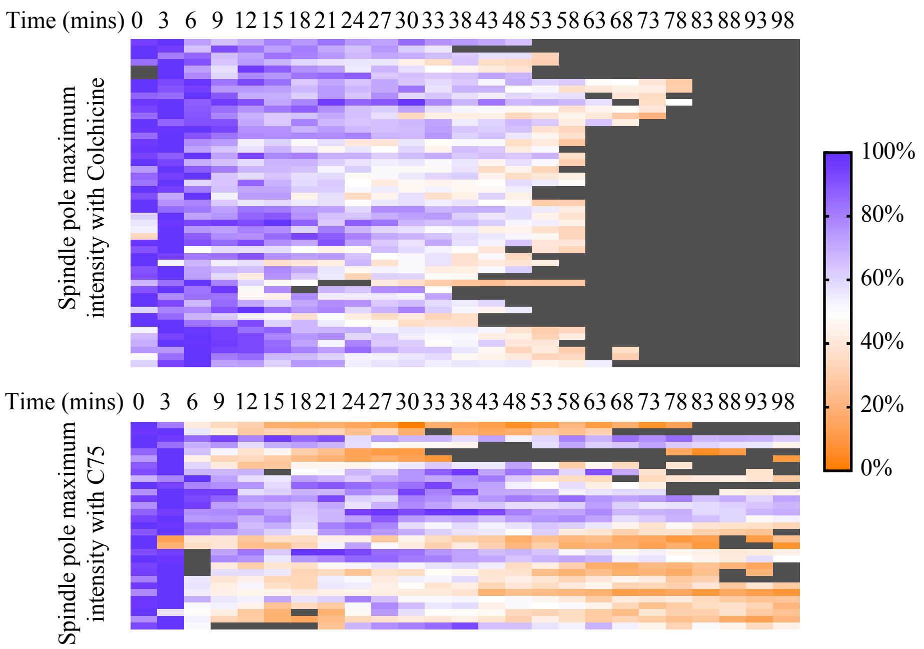

Supplement: Supplementary file 1 — Supplementary Information. [file 41598_2021_3076_MOESM1_ESM.pdf]
